# Viniferin-Rich Phytocomplex from *Vitis vinifera* L. Plant Cell Culture Mitigates Neuroinflammation in BV2 Microglia Cells

**DOI:** 10.3390/molecules31010196

**Published:** 2026-01-05

**Authors:** Giacomina Videtta, Chiara Sasia, Sofia Quadrino, Oriana Bertaiola, Chiara Guarnerio, Elisa Bianchi, Giacomo Biagiotti, Barbara Richichi, Stefano Cicchi, Giovanna Pressi, Nicoletta Galeotti

**Affiliations:** 1 Department of Neurosciences, Psychology, Drug Research and Child Health (Neurofarba), Section of Pharmacology and Toxicology, Laboratory of Neuroinflammation and Cell Senescence, University of Florence, Viale G. Pieraccini 6, 50139 Florence, FI, Italy; giacomina.videtta@unifi.it (G.V.); chiara.sasia@unifi.it (C.S.); sofia.quadrino@unifi.it (S.Q.); 2 Aethera Biotech Srl, Via Dell’Innovazione 1, 36043 Camisano Vicentino, VI, Italy; orianabertaiola@aetherabiotech.it (O.B.); chiaraguarnerio@aetherabiotech.it (C.G.); giovannapressi@aetherabiotech.it (G.P.); 3 Department of Chemistry “Ugo Schiff,” University of Florence, Via della Lastruccia 3–13, 50019 Sesto Fiorentino, FI, Italy; elisa.bianchi1@unifi.it (E.B.); giacomo.biagiotti@unifi.it (G.B.); barbara.richichi@unifi.it (B.R.); stefano.cicchi@unifi.it (S.C.)

**Keywords:** *Vitis vinifera* L., neuroinflammation, microglia, BV2 cells, plant cell culture, cellulose nanocrystal, nanoformulation

## Abstract

Activation of microglia and resulting neuroinflammation are central processes that significantly contribute to neurodegenerative disease progression. Treatments capable of attenuating neuroinflammation are therefore an urgent medical need. *Vitis vinifera* L., cultivated since ancient times for its fruits, is known for its antioxidant and anti-inflammatory activities. However, polyphenols, the main bioactive molecules in *V. vinifera* extracts, exhibit considerable variability due to numerous hard-to-control factors, which complicates the production of standardized extracts with consistent biological activity. To address this issue, plant cell culture biotechnology was used to produce a highly standardized *V. vinifera* phytocomplex (VP), and its anti-neuroinflammatory profile was investigated in LPS-stimulated microglial cells, an in vitro model of neuroinflammation. VP reduced the LPS-induced pro-inflammatory phenotype, improved cell viability and cell number, attenuated NF-κB activation and ERK1/2 phosphorylation, and increased SIRT1 levels. To overcome VP’s poor water solubility, water-soluble cellulose nanocrystal (CNC)-based formulations were developed and tested. VP-CNC formulations markedly reduced the BV2 pro-inflammatory phenotype and increased cell viability under both basal and LPS-stimulated conditions. The nanoformulations also decreased pERK1/2 levels and increased SIRT1 expression, exhibiting biological activities comparable to VP alone. *V. vinifera* phytocomplex derived from plant cell cultures represents an innovative and standardized product with promising anti-neuroinflammatory properties.

## 1. Introduction

Neuroinflammation is a sustained inflammatory response within the central nervous system (CNS) that refers to an infiltration of immune cells into the CNS [1] and to the activation of CNS resident immune cells, primarily microglia and astrocytes, and non-immune cell types [2,3]. These cells release proinflammatory mediators and reactive oxygen and nitrogen species (ROS and RNS) that initiate and propagate neuroinflammatory responses. Acute neuroinflammation elicits a transient, self-limiting reaction that promotes tissue repair and can serve protective functions. When unresolved, however, the inflammatory cycle becomes prolonged, leading to chronic neuroinflammation, which is ultimately detrimental to the CNS [3,4].

The immune response is initially triggered by microglial activation. While microglial activity is critical for maintaining brain homeostasis [5,6,7], excessive or prolonged activation leads to the massive release of proinflammatory mediators that promote CNS damage and influence disease outcomes and pathology [8,9]. Neuroinflammation and microglial activation are central events driving neurodegeneration and have been recognized as major contributors to the progression of several neurodegenerative conditions [2,8,10].

*Vitis vinifera* L. (grape), cultivated for thousands of years by many civilizations, represents one of the largest fruit crops used for wine, juice, and fresh consumption. Its most important active constituents are phenolic compounds, mainly phenolic acids, flavonoids, proanthocyanidins, and characteristic stilbene derivatives [11]. However, the polyphenol profile of *Vitis vinifera* L. is highly complex, with concentrations that vary markedly according to the morphological part analyzed [12]. It is also influenced by factors such as variety, maturity, post-harvest storage, and environmental parameters including location, light conditions, temperature, nutrition, water availability, microorganisms, and viticultural practices [13], making it difficult to standardize grape-based products.

*Vitis vinifera* L. has traditionally been used as a laxative and carminative, and as a remedy for colds and flu, wound care, allergies, and bronchitis [14,15]. Experimental studies have shown that bioactive compounds found in grapes exhibit antioxidant, antibacterial, antifungal, antidiabetic, anticancer, and cardioprotective activities [16]. The antioxidant properties of grape polyphenols have also been described within the CNS, where they exert beneficial neuroprotective effects [17,18]. These findings suggest that *Vitis vinifera* L. may play a key role in attenuating neuroinflammation, with potential benefits for neurodegenerative diseases. However, the pharmacological effects of *Vitis vinifera* L. on activated microglia have not been elucidated. Thus, our study aimed to evaluate its modulatory effects on microglia-mediated neuroinflammation in LPS-stimulated microglia.

A promising strategy for generating uniform, contaminant-free plant materials on an industrial scale is the use of in vitro plant cell culture technology. By cultivating plant cells under strictly regulated environmental conditions [19], this method ensures consistent production of bioactive metabolites and avoids the fluctuations typical of conventional plant extracts. Extracts and phytocomplexes produced in this way can be accurately standardized for both primary and secondary metabolites and consistently meet safety requirements due to their absence of contaminants and phytochemical uniformity [20].

Furthermore, this approach contributes to biodiversity conservation and promotes environmental sustainability by significantly reducing the use of natural resources, including water and soil. It also eliminates seasonal and geographical limitations and improves consumer safety by preventing contamination from heavy metals, pesticides, aflatoxins, and microbial agents, while providing a high degree of standardization.

Our results indicated that a viniferin-rich *Vitis vinifera* L. phytocomplex (VP) produced through plant cell culture suppressed the proinflammatory morphology and restored cell viability in LPS-stimulated BV2 microglial cells by inhibiting NF-κB activation and increasing SIRT1 expression. Furthermore, the action of VP is made more efficient using a formulation with cellulose nanocrystals (CNC) derivatives, which enhance the dispersibility of the phytocomplex in water.

## 2. Results

### 2.1. Characterization of Vitis vinifera L. Standardized Phytocomplex Obtained from Cell Culture Suspensions

#### 2.1.1. Development and Preparation of *V. vinifera* L. Standardized Phytocomplex (VP)

**Figure 1:**
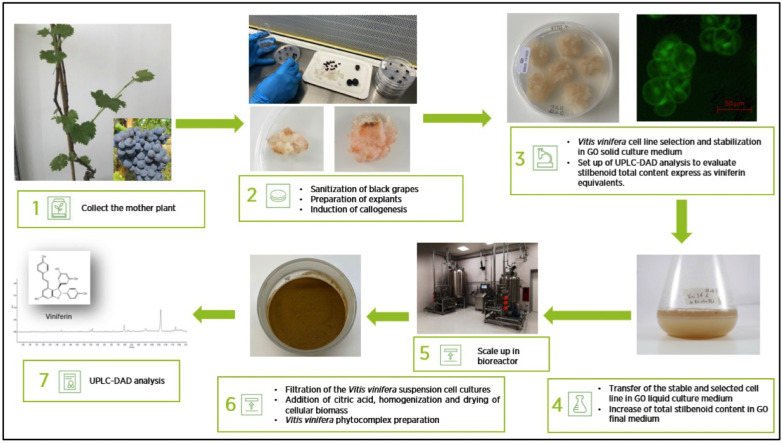
Flow chart of the development and preparation of VP.

A stable, selected *V. vinifera* L. cell line was generated from black grapes using G0 solid medium (Gamborg B5 supplemented with 20 g/L sucrose, 0.8% (*w*/*v*) plant agar, 1 mg/L NAA, 1 mg/L IAA, 1 mg/L K, and a final pH of 6.5). Figure 1 shows the flow chart for the development and preparation of the standardized *V. vinifera* phytocomplex (VP). After six months of routine subculturing on the same medium, the cells exhibited a pale yellow coloration, a friable texture, and a rapid growth rate, requiring transfer to fresh G0 solid medium every three weeks. The appearance of the stabilized V. vinifera cell line grown on G0 solid medium is shown in Figure 1. Fluorescein diacetate staining revealed both the morphology and viability of the plant cells maintained under these conditions (Figure 1). Optimization of total stilbenoid production in *V. vinifera* suspension cultures was achieved using a G0 liquid medium enriched with a higher sucrose concentration (40 g/L) and supplemented with 7.5 mg/L methyl jasmonate and 25 mM β-cyclodextrin, added three days after fermentation began. Figure 1 also illustrates the appearance of the suspension culture in the optimized liquid medium. Cells cultivated in this medium for 14 days were harvested for phytocomplex preparation.

#### 2.1.2. UPLC-DAD and LC-MS Analysis

To characterize the secondary metabolites present in the standardized *Vitis vinifera* phytocomplex (VP), a complementary analytical strategy combining UPLC–DAD and LC–MS was employed.

**Figure 2:**
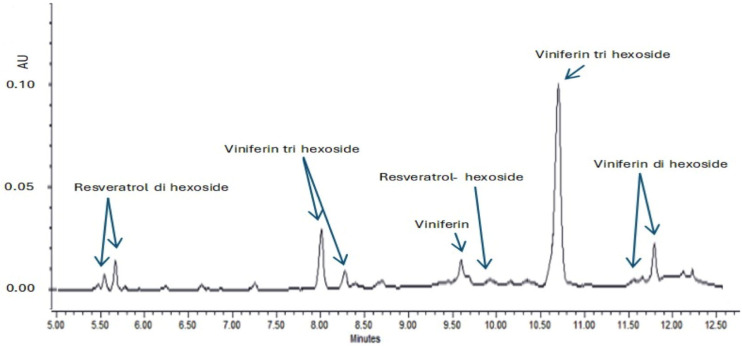
Representative UPLC-DAD chromatogram of VP recorded at 330 nm.

UPLC–DAD analysis performed at 330 nm revealed several closely eluting peaks between 5.5 and 18.0 min with similar UV–Vis absorption maxima (320–325 nm), characteristic of stilbenoid derivatives (Figure 2). Total stilbenoid content, expressed as (+)-ε-viniferin equivalents, was quantified as 0.15 ± 0.02% (*w*/*w*). The main stilbenoid classes detected included viniferin hexosides, viniferin di-hexosides, viniferin tri-hexosides, and resveratrol di-hexosides.

LC–MS analysis provided detailed qualitative information on VP composition. MS analysis in negative ion mode revealed a dominant diagnostic fragment at *m*/*z* 227, consistent with resveratrol-derived stilbenoid structures. Several glycosylated stilbenoids and viniferin oligomers were detected, together with additional peaks tentatively assigned to stilbenoid-related structures based on their fragmentation behavior, although their exact structures could not be fully elucidated. Compounds were mainly detected as deprotonated molecules ([M–H]^−^), showing sequential neutral losses of hexose units (−162 Da). Free resveratrol was not detected, as *m*/*z* 227 was observed only as a fragment and not as a precursor ion. Minor discrepancies in elution order between UPLC–DAD and LC–MS analyses were observed and attributed to differences in chromatographic configuration and MS-compatible conditions. Compound identification relied on accurate mass measurements, MS/MS fragmentation, UV spectra, and literature comparison rather than retention time alone. Identified and tentatively annotated compounds are summarized in Table S1.

### 2.2. Effect of VP on an In Vitro Model of Neuroinflammation

#### 2.2.1. Suppression of Microglia Proinflammatory Phenotype by VP

**Figure 3:**
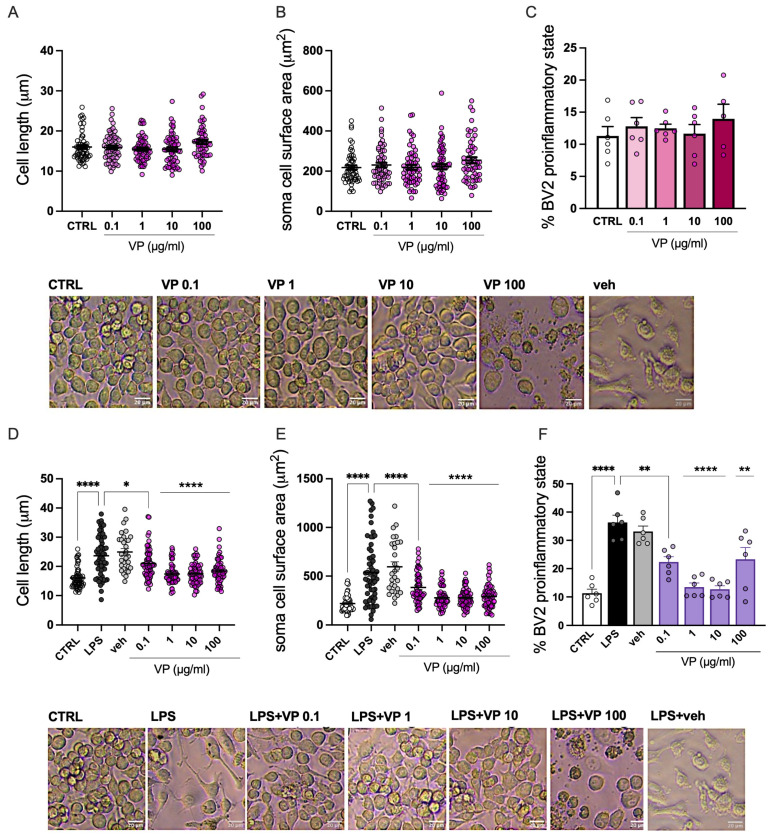
Morphological analysis of microglia cells following VP treatment. No variation was observed on BV2 cell diameter (**A**), soma surface area (**B**), and percentage of cells in the proinflammatory state (**C**) with VP treatment (0.1–100 µg/mL) at steady state. Dose-dependent attenuation by VP of LPS-induced increase in diameter (**D**), soma surface area (**E**), and percentage of cells in the proinflammatory state (**F**). Representative images of VP-treated unstimulated and stimulated BV2 cells. Scale bar: 20 µm. LPS: 250 ng/mL for 24 h. Veh = vehicle (50% DMSO). * *p* < 0.05, ** *p* < 0.01, **** *p* < 0.0001 vs. LPS.

Dose-dependent studies of VP (0.1–100 µg/mL) on BV2 morphology were performed by analyzing cell length (Figure 3A), soma area (Figure 3B), and the percentage of cells in the proinflammatory state (Figure 3C) under resting conditions. At all concentrations tested, VP did not significantly modify cell morphology compared with the CTRL group.

BV2 cells exhibit distinct morphological phenotypes in resting versus proinflammatory states. Morphological analysis of BV2 cells under LPS stimulation showed a shift toward a stretched and elongated phenotype, as demonstrated by a marked increase in cell length and soma area. VP (0.1–100 µg/mL) reduced cell length (Figure 3D) and cell surface area (Figure 3E). Finally, VP reduced the percentage of cells displaying the elongated proinflammatory phenotype (Figure 3F).

#### 2.2.2. Effect of VP on Microglia Cell Viability

**Figure 4:**
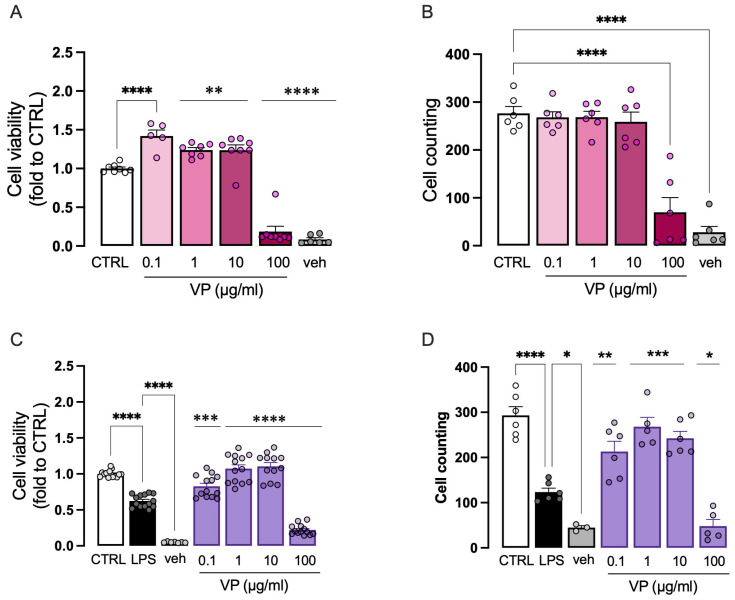
Effect of VP on cell viability. (**A**) VP (0.1–10) increases cell viability at resting conditions. VP 100 and veh drop of cell viability. (**B**) Lack of effect on cell number by VP (0.1–10) at steady state and reduction in cell count by VP 100 and veh. ** *p* < 0.01 and **** *p* < 0.0001 vs. CTRL. LPS-stimulated cells showed reduced cell viability (**C**) and number (**D**). Dose-dependent reversal of LPS-induced effect by VP at 0.1–10 was observed, whereas VP 100 showed toxic activity comparable to veh. LPS: 250 ng/mL for 24 h. Veh = vehicle (50% DMSO). * *p* < 0.05, ** *p* < 0.01, *** *p* < 0.001, **** *p* < 0.0001 vs. LPS.

The effect of VP on BV2 cell viability was investigated in the absence and presence of LPS stimulation. Under basal conditions, VP significantly increased cell viability at doses of 0.1, 1, and 10 µg/mL. At the dose of 100 µg/mL, there was a drastic reduction in cell viability, likely due to the amount of DMSO required to dissolve the phytocomplex (Figure 4A). The effect of VP on cell number was also assessed, showing no effect at doses ranging from 0.1 to 10 µg/mL. Consistent with the cell viability data, the 100 µg/mL dose caused a marked reduction (Figure 4B).

Exposure of BV2 cells to LPS (250 ng/mL for 24 h) significantly reduced both cell viability (Figure 4C) and cell number (Figure 4D). Doses of 0.1, 1, and 10 µg/mL dose-dependently restored cell viability and mitigated the reduction in cell number. The 100 µg/mL dose caused a drastic decrease in both parameters, again due to the DMSO content. Therefore, the 100 µg/mL dose was excluded from further analyses.

#### 2.2.3. Modulation of Neuroinflammation Markers by VP

**Figure 5:**
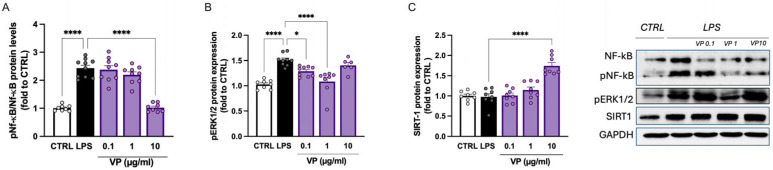
VP-induced modulation of neuroinflammation biomarkers. Dose-dependent reduction in LPS-induced NF-kB activation (**A**) and ERK1/2 over-phosphorylation (**B**). VP-induced increase in SIRT1 protein levels (**C**). LPS: 250 ng/mL for 24 h. * *p* < 0.05 and **** *p* < 0.0001 vs. LPS.

LPS stimulation was associated with robust activation of the NF-κB pathway, as demonstrated by the increased expression of phosphorylated NF-κB (p-NF-κB). In the LPS-treated group, the p-NF-κB/NF-κB ratio was more than twice that of the control group. Doses of 0.1 and 1 µg/mL had no effect, whereas treatment with VP at 10 µg/mL restored the p-NF-κB/NF-κB ratio to basal levels (Figure 5A).

Microglial cells exposed to LPS also showed increased phosphorylation of the MAPK ERK1/2. VP at 0.1 and 1 µg/mL dose-dependently attenuated p-ERK1/2 overactivation, with a peak effect observed at 1 µg/mL. This effect was reduced at higher doses (Figure 5B).

Finally, the effect of VP on SIRT1 levels was assessed. LPS-stimulated cells showed SIRT1 levels comparable to the control group. VP at 0.1 and 1 µg/mL had no effect, whereas treatment with 10 µg/mL resulted in a marked increase in SIRT1 protein expression (Figure 5C).

### 2.3. Pharmacological Profile of VP Nanocellulose Formulations

#### 2.3.1. Analysis of VP-CNC Formulations

**Scheme 1:**
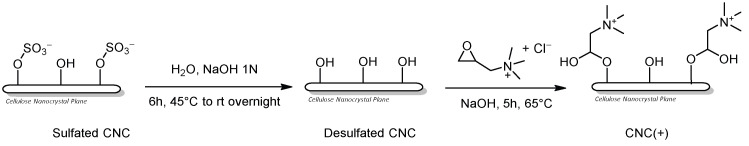
Synthesis of CNC (+).

**Figure 6:**
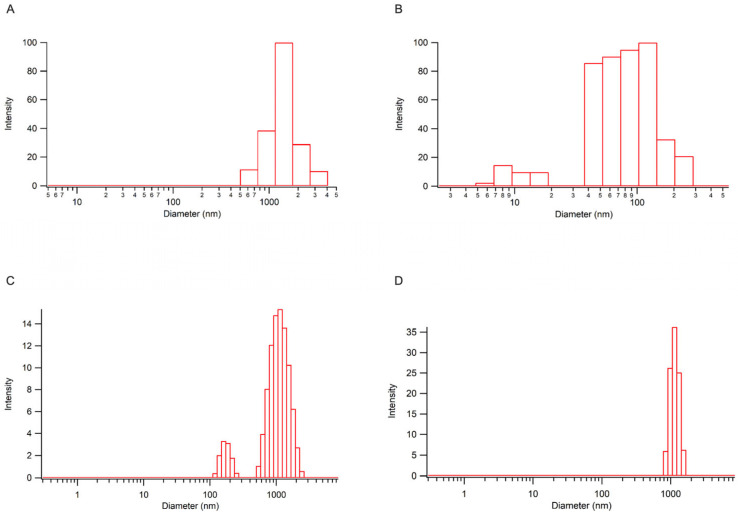
DLS column chart of CNC(+) (**A**), CNC(−) (**B**), VP-CNC(+) 1:10 (**C**), and VP-CNC (−) 1:10 (**D**).

VP showed promising anti-neuroinflammatory properties. However, the amount of DMSO required to dissolve the phytocomplex made it impossible to investigate concentrations higher than 10 µg/mL. To overcome the poor water solubility of VP and avoid any potential confounding effect produced by the vehicle, we used water-dispersible cellulose nanocrystal formulations as innovative drug delivery systems. This choice is justified by the well-known amphiphilic nature of cellulose nanocrystals (from now on referred to as CNC) [21,22], which have already been applied in the delivery of poorly soluble drugs [23,24]. Specifically, two distinct nanoformulations were used: VP–cellulose nanocrystals (+) (VP-CNC(+) for its syntesis see Section 4.3 and Scheme 1)) and VP-sulfated cellulose nanocrystals (−) (VP-CNC(−)) containing VP and CNC in a 1:10 ratio. The two formulations were prepared using a ball milling procedure [25] by employing experimental conditions previously reported with minor modifications [26,27,28]. Commercially available sulfated cellulose nanocrystals (CNC (−), ζ-potential: (−50.51 ± 2.97) mV) were used as starting material for the preparation of the CNC(+) (ζ-potential: (48.91 ± 3.81) mV) by employing a previously reported protocol with minor modifications (for its preparation, see Section 4.3 in the Materials and Methods Section). Figure 6 shows the distribution of nanoparticle size, obtained by dynamic light scattering (DLS), for both cellulose nanocrystals (i.e., CNC (−) and CNC (+)) and the corresponding nanoformulations (VP-CNC(+) and VP-CNC(−)).

#### 2.3.2. Attenuation of Proinflammatory Morphology by VP-CNC Nanoformulations

**Figure 7:**
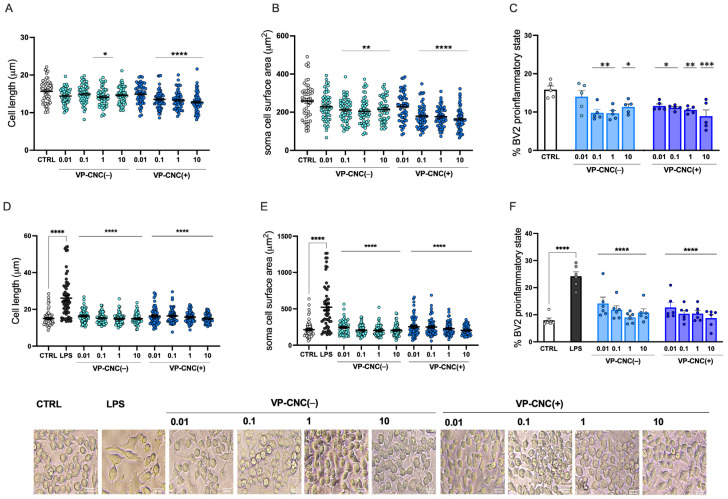
Morphological analysis of microglia cells following VP-CNC treatment. Reduction in BV2 cell diameter (**A**), soma surface area (**B**), and percentage of cells in the proinflammatory state (**C**) by VP-CNC formulations (0.01–10 µg/mL) at basal conditions. * *p* < 0.05, ** *p* < 0.01, *** *p* < 0.001, **** *p* < 0.0001 vs. CTRL. VP-CNC attenuation of LPS-induced increase in diameter (**D**), soma surface area (**E**), and percentage of cells in the proinflammatory state (**F**). Representative images of VP-CN-treated unstimulated and stimulated BV2 cells. Scale bar: 20 µm. LPS: 250 ng/mL for 24 h. Concentrations refer to the amount of VP contained in each formulation (VP-CNC 1:10). **** *p* < 0.0001 vs. LPS.

Microglia are heterogeneous cells that, even under resting conditions, include a proportion of cells (approximately 15%) in the proinflammatory state. Dose–response curves for the VP-CNC(−) and VP-CNC(+) formulations showed a significant reduction in cell diameter (Figure 7A) and soma area (Figure 7B) under basal conditions, starting from 0.1 µg/mL. Furthermore, treatment with VP-CNC(−) and VP-CNC(+) reduced the percentage of cells in the proinflammatory state (Figure 7C).

Morphological analysis of LPS-stimulated cells demonstrated that both formulations effectively reduced the LPS-induced increase in cell diameter at all doses tested (Figure 7D) and normalized the soma area (Figure 7E) to basal levels. Both formulations also reduced the LPS-induced increase in the percentage of cells in the proinflammatory state, restoring it to values comparable to the control group (Figure 7F).

#### 2.3.3. Improvement of Microglia Cell Viability by VP Nanoformulations

**Figure 8:**
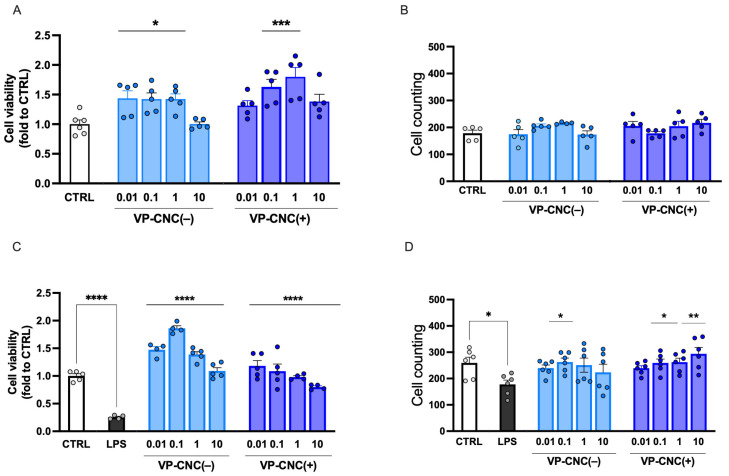
Effect of VP-CNC on cell viability. (**A**) VP-CNC(−) (0.01–1) and VP-CNC(+) (0.1–1) increase cell viability at resting conditions. (**B**) Lack of effect on cell number by VP-CNC(−) and VP-CNC(+) (0.01–10) at steady state. * *p* < 0.05, *** *p* < 0.001 vs. CTRL. LPS-stimulated cells showed reduced cell viability (**C**) and number (**D**). Dose-dependent reversal of LPS-induced effect by VP-CNC(−) and VP-CNC(+) 0.01–10. LPS: 250 ng/mL for 24 h. Concentrations refer to the amount of VP contained in each formulation (VP-CNC 1:10). * *p* < 0.05, ** *p* < 0.01, **** *p* < 0.0001 vs. LPS.

VP-CNC(−) and VP-CNC(+) dose-dependently increased cell viability under resting conditions, with a peak effect observed at 10 µg/mL, corresponding to VP 1 µg/mL (Figure 8A). Both treatments did not affect cell number under basal conditions (Figure 8B).

The nanocellulose formulations were also effective under proinflammatory conditions. Specifically, VP-CNC(−) and VP-CNC(+) restored cell viability reduced by LPS exposure (Figure 8C) and increased cell number to levels comparable to the control group (Figure 8D).

#### 2.3.4. Effect of VP-CNC Nanoformulation on pERK1/2 and SIRT1 Levels

**Figure 9:**
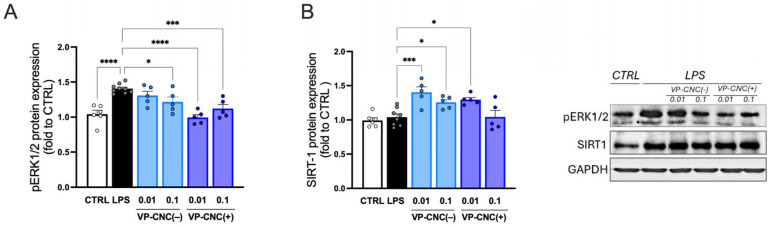
Effect of VP-CNC on neuroinflammation biomarkers. (**A**) Dose-dependent reduction in LPS-induced ERK1/2 over-phosphorylation. (**B**) VP-CNC increased SIRT1 protein levels. Concentrations refer to the amount of VP contained in each formulation (VP-CNC 1:10). * *p* < 0.05, *** *p* < 0.001, **** *p* < 0.0001 vs. LPS.

To evaluate the efficacy of the CNC formulations on neuroinflammation markers, we assessed their effects on the expression of p-ERK1/2 (Figure 9A) and SIRT1 (Figure 9B). Treatment of LPS-stimulated cells with the highest effective doses of the formulations reduced p-ERK1/2 over-phosphorylation and increased SIRT1 levels. These results demonstrate that the VP-CNC formulations were comparably effective to unformulated VP. Importantly, these findings indicate the absence of confounding effects from the vehicle at the low VP doses and confirm CNC as a suitable drug delivery system for plant cell culture-derived phytocomplexes.

## 3. Discussion

The levels of bioactive molecules in *V. vinifera* extracts vary widely due to numerous difficult-to-control factors, including seasonality, genetic differences among grape varieties, plant age, cultivation area, and the specific tissues used to produce the extracts [7,8]. This variability makes it challenging to obtain standardized *V. vinifera* derivatives with reproducible metabolite profiles. The considerable phytochemical variability of plant-derived extracts can also reduce their effectiveness. To overcome these limitations and ensure reproducibility and efficacy of biological activities, the present study utilized in vitro plant cell culture biotechnology. We investigated the pharmacological profile of a plant cell-derived *V. vinifera* L. phytocomplex in a model of microglia-mediated neuroinflammation.

Microglia support brain homeostasis during steady-state conditions [29]. Therefore, we first investigated the effects of VP on resting microglial cells. Treatment, while not altering the resting microglial phenotype, increased cell viability, suggesting a potential CNS-protective effect. In addition to their homeostatic role, microglia play a key role during injury or inflammatory insults, where their activation is intended to protect the CNS by promoting the release of inflammatory mediators and activating tissue repair mechanisms. However, prolonged and uncontrolled microglial activation can sustain neuroinflammation, exacerbating neuronal damage [30]. Accordingly, we tested the efficacy of VP in an in vitro model of neuroinflammation based on LPS stimulation of BV2 microglial cells [31]. Microglia are dynamic cells that, under inflammatory conditions, change their morphology from a resting phenotype to a proinflammatory phenotype. Morphological analysis showed that VP reversed the LPS-induced shift in BV2 cells from a short, round morphology to an elongated, large-sized phenotype. VP also restored the reduced cell viability and cell number under LPS stimulation, providing an initial indication of its anti-neuroinflammatory effect. These results are consistent with previous studies demonstrating anti-inflammatory effects of *Vitis vinifera* L. leaf extract in human keratinocytes [32] and murine macrophages [33] exposed to proinflammatory stimuli (i.e., tumor necrosis factor-α and LPS).

Activated microglia development and maintenance depend on constant engagement of colony-stimulating factor 1 receptor (CSF1R), a receptor tyrosine kinase that transmits intracellular signals, such as activation of protein kinase B (AKT) and extracellular signal-regulated kinases (ERKs), which promote microglial proliferation and survival [34]. In our model, neuroinflammation was induced in BV2 microglial cells by LPS exposure. LPS, by stimulating toll-like receptor 4 (TLR4), activates downstream MAPK signaling, which promotes the synthesis of proinflammatory mediators [35]. MAPKs are also involved in NF-κB activation, a transcription factor that drives microglial activation. Previous studies have reported that NF-κB is activated by MAPK ERK1/2 through mechanisms such as phosphorylation of NF-κB inhibitory factor IκBα and nuclear translocation of p65, contributing to a proinflammatory response [36,37]. VP reduced NF-κB activation and ERK1/2 hyperphosphorylation, further supporting its anti-neuroinflammatory mechanism.

Silent information regulator sirtuin 1 (SIRT1), a deacetylase member of the sirtuin family, regulates various biological functions and plays a key role in modulating inflammation [38]. Studies suggest that SIRT1 has strong anti-inflammatory effects by inhibiting the expression of factors involved in inflammatory pathways [38], including the NF-κB p65 subunit, thus inhibiting NF-κB activity [39]. SIRT1 may also limit NF-κB nuclear translocation and its DNA-binding ability [40]. Numerous anti-inflammatory drugs act by upregulating SIRT1 expression [41,42]. VP markedly increased microglial SIRT1 levels, consistently with an anti-neuroinflammatory activity. No significant variation in SIRT1 expression was observed following LPS exposure. This seemingly contradictory result may reflect the variability and context-dependent regulation of SIRT1 levels during inflammatory conditions [43].

The therapeutic effects of *V. vinifera* are attributed to its active constituents, primarily stilbenes, a class of polyphenolic compounds. In particular, resveratrol and viniferin, a resveratrol dimer, have been extensively studied for their antioxidant and anti-inflammatory properties. Their anti-inflammatory mechanisms are mainly associated with inhibition of both NF-κB activation and MAPK hyperphosphorylation [44]. Furthermore, resveratrol is a well-known SIRT1 activator [45,46]. Evidence also suggests that viniferins increase SIRT1 expression in vascular endothelial cells [47], while in adipocytes, ε-viniferin raises SIRT1 expression more effectively than resveratrol [48]. Thus, the pharmacological activity of VP appears to be related to its stilbenoid content, likely driven predominantly by viniferin.

Studies have described ε-viniferin as poorly bioavailable, similar to other monomeric and dimeric stilbenes [49]. Despite this unfavorable property, multiple lines of evidence have demonstrated its biological activity [50], consistent with the present findings. However, the poor water solubility of VP may limit its potential clinical application due to the need for lipophilic solvents, which can induce cellular toxicity. To address this limitation, an innovative nanoformulation was employed.

Drug delivery systems have received considerable attention over the past decade because they offer several potential advantages, including reduced side effects, improved therapeutic efficacy, and lower effective doses [51]. Cellulose, a major natural plant component, possesses excellent renewability and biodegradability, making it a suitable, natural, non-toxic, and inexpensive material while maintaining good biological activity with minimal side effects. Technological advances have generated significant interest in different types of nanocelluloses [52], which have emerged as promising “green” materials for drug delivery applications [53].

We developed and investigated a water-dispersible, cellulose nanocrystal-based formulation containing VP that displayed biological activity comparable to unformulated VP, without inducing cellular toxicity. Moreover, the improved solubility enabled pharmacological effects at doses ten times lower than those required for the unformulated phytocomplex, indicating that this delivery system enhances the solubility and efficacy of natural compounds.

In conclusion, *V. vinifera* phytocomplex obtained from cell culture suspensions represents an innovative and standardized ingredient with a strong safety profile, supported by a sustainable and clean production process. Plant cell culture technology ensures controlled growth conditions for the selected and stable *V. vinifera* cell line, guaranteeing a high degree of standardization in the phytocomplex composition. This consistency in bioactive molecule content translates into reproducible biological efficacy.

Future studies will further explore the pharmacological activity of VP in models of neuroinflammation-mediated pathological conditions to assess its potential for clinical translation. Additionally, it will be important to dissect the contribution of individual constituents to identify the main active component(s) and determine whether synergistic interactions among VP stilbenoids enhance their overall biological effect.

## 4. Materials and Methods

### 4.1. Vitis vinifera L. Phytocomplex (VP) from Cell Culture Suspensions

A standardized and sustainable phytocomplex of *Vitis vinifera* L. (VP) was developed using plant cell culture technology. *V. vinifera* young plant was bought and certified from the nursery plant “Vivai Busatta”, Camisano Vicentino, Vicenza, Italy. A stabilized and selected cell line specified on the biosynthesis of stilbenoids was obtained by dissected *V. vinifera* fruits (black grapes) using the following treatments in sequence: 70% (*v*/*v*) ethanol (Honeywell, Wunstorfer Straβe 40, D-30926, Seelze, Germany) in water for about 1 min, followed by washing with sterile distilled water for 3 min. They were then washed with 2% (*v*/*v*) sodium hypochlorite solution (6–14% active chlorine, Merck KGaA, Darmstadt, Germany) and 0.1% (*v*/*v*) Tween 20 (Duchefa, Haarlem, The Netherlands) for 3 min. Finally, they were washed at least 3 times with sterile distilled water for 5 min each. After sanitization, the black grapes were cut into small pieces (explants) of sub-centimetric dimensions. The sanitized fragments of black grapes were deposited in several Petri dishes contained Gamborg B5 medium [54] supplemented with 20 g/L sucrose (Sudzucker AG, Manheim, Germany), 1 mg/L of naphthalenacetic acid (NAA) (Duchefa), 1 mg/L of indolacetic acid (IAA) (Duchefa), 1 mg/L of Kinetin (K) (Duchefa), and 0.8% *w*/*v* of plant agar (Duchefa), and the pH was adjusted to 6.5 (G0 solid medium).

Petri dishes containing explants were incubated at 25 ± 1 °C in the dark. Calli were grown after 20 days of incubation and were subjected to subculture for 6 months (3 weeks of each subculture) until they became friable and homogeneous, with a constant growth rate (selected and stable *V. vinifera* cell line). The suspension cultures were obtained by transferring a part of selected calli (10% *w*/*v*) into 100 mL Erlenmeyer flasks containing 20 mL of G0 liquid culture medium (G0 without plant agar). The suspension cultures were incubated in a climatic growth room in dark conditions at 25 ± 2 °C on a rotary shaker in constant agitation at 120 rpm and were subcultivated in larger volume (from 0.1 L flasks to 3 L flasks) every 7 days of fermentation. To produce large quantities of biomass, the cultured suspension was transferred and adapted to growth in a bioreactor of progressively increasing size (5 L and 13 L volume) with an amount of cell suspension inoculated into the liquid medium equal to 7% *v*/*v*.

To increase the total stilbenoid content, expressed as (+)-ε-viniferin equivalent, after 7 days of fermentation in G0 liquid medium, 7% (*v*/*v*) of *V. vinifera* cell suspension was transferred to a final liquid medium (G0 containing 40 g/L of sucrose). After 3 days of fermentation, 7.5 mg/L methyl jasmonate (Merck K GoA Darmstadt, Germany) and 25 mM β-cyclodextrin (Merck K GoA Darmstadt, Germany) were added to the suspension cell cultures.

After 14 days of growth in G0 final liquid medium at 25 ± 2 °C in a climatic growth room in the dark and on a rotary shaker in constant agitation at 120 rpm, the *V. vinifera* suspension cell cultures were filtered by a 50 µm mesh filter to remove liquid culture medium. The collected cells were washed with twice the volume of saline solution (0.9% *w*/*w* NaCl in sterile water), citric acid 1% (*w*/*w*) was added, and then the cells were homogenized with an Ultra Turrax homogenizer at 15,000 rpm for 20 min. The biomass of homogenized cells was dried using a Mini Spray Dryer (BUCHI-B290) to obtain a powder of *V. vinifera* standardized phytocomplex (VP) with a high content of total stilbenoids expressed as (+)-ε-viniferin equivalent.

### 4.2. UPLC-DAD and LC-MS Analysis of Vitis vinifera L. Standardized Phytocomplex (VP)

#### 4.2.1. Sample Preparation

Preliminary extraction experiments were conducted to identify the most suitable solvent system for the recovery of phenolic compounds, with particular emphasis on stilbenoids. Methanol–water (50:50, *v*/*v*) was identified as the most efficient extraction solvents. For routine UPLC–DAD analysis, VP (25 mg) was dissolved rather than exhaustively extracted in methanol–water (50:50, *v*/*v*). The suspension was vortexed for 30 s and sonicated for 15 min in an ice bath to ensure complete dissolution. Samples were then centrifuged at 13,000 rpm for 10 min at 6 °C. The supernatant was diluted to a ratio of 1:10 with the same solvent mixture and filtered through a 0.22 µm membrane prior to injection. Four independent preparations were performed to assess reproducibility.

For LC–MS analysis, a more concentrated methanol–water (50:50, *v*/*v*) solution of VP was analyzed to enhance MS sensitivity toward minor components.

#### 4.2.2. UPLC–DAD Conditions

UPLC–DAD analyses were performed on an Acquity UPLC system (Waters, Milford, MA, USA) equipped with an Acquity UPLC BEH C18 column (1.7 μm, 2.1 × 100 mm) and a VanGuard BEH C18 pre-column (1.7 μm, 2.1 × 5 mm). The system included a Binary Solvent Manager I-Class, a Sample Manager-FTN I-Class autosampler, and a PDA eλ detector. Data acquisition and processing were performed using Empower 3 software (Waters). The mobile phase consisted of solvent A (water with 0.1% formic acid) and solvent B (acetonitrile with 0.1% formic acid). The run started at 90% solvent A with a constant flow rate of 0.2 mL/min. Column temperature was maintained at 30 °C.

Quantification of total stilbenoids was performed by monitoring chromatograms at 330 nm, a wavelength characteristic of stilbenoid absorption. Total stilbenoid content was calculated using an external calibration curve prepared with a commercial (+)-ε-viniferin standard (purity ≥ 97.2%, Extrasynthase, Genay, France). Results were expressed as (+)-ε-viniferin equivalents. Data acquisition and processing were performed using Empower 3 software.

#### 4.2.3. LC–MS Analysis

LC–MS analysis was carried out to achieve qualitative characterization of VP constituents and to support compound annotation. LC–MS profiling was carried out on an LC system coupled to an electrospray ionization (ESI) source coupled to an ion trap analyzer (Agilent Technologies Italia S.p.A., Cernusco sul Naviglio, Italy). Chromatographic separation was performed using an Agilent SB C18 column (4.6 × 50 mm, 1.8 μm). The mobile phases consisted of A, water containing 0.1% formic acid; B, acetonitrile; and C, methanol. The flow rate was set at 0.75 mL/min, and the total run time was 30 min. The LC system was equipped with a diode array detector (DAD) positioned upstream of the mass spectrometer. DAD spectra were acquired in the range of 190–600 nm, and chromatograms were monitored at 330 nm (stilbenoids), 350 nm (flavonols), and 254 nm (generic aromatic compounds) to support compound classification. Mass spectrometric detection was performed in both positive and negative ion modes, with negative ion mode preferentially employed for phenolic compounds. Full-scan mass spectra were acquired over an *m*/*z* range of 100–1500, and MS^n^; fragmentation experiments were performed using data-dependent acquisition (collision energy was set according to instrument default parameters) to support compound annotation. Compound identification was based on a multi-parameter approach combining retention times, UV–Vis spectra, accurate *m*/*z* values, MS/MS fragmentation patterns, comparison with reference standards when available, and literature data.

### 4.3. Synthesis CNC (+)

We followed the procedure used by Liu, Y. et al. [55] with minor changes.

Sodium hydroxide powder (400 mg) was added to a suspension of sulfated cellulose nanocrystals (sulfated CNC, commercially available CelluForce NCC^®^ NCV100-NASD90; 500 mg, 5 wt%) in water (10 mL) to obtain a 1 N NaOH solution. The reaction mixture was stirred at 65 °C for 6 h and subsequently kept at room temperature overnight. After dialysis against Milli-Q water until neutral pH was reached, the dispersion was concentrated to obtain a suspension of desulfated CNC (424 mg, 8.2 wt%) in water. Next, NaOH (21 mg, 5% by weight of CNC) was added, and the reaction mixture was stirred for 30 min at room temperature. Glycidyltrimethylammonium chloride (894 mg, 5.9 mmol) was then added in a molar ratio of 2.5:1 with respect to desulfated CNC. The reaction mixture was stirred at 65 °C for 5 h. Finally, the mixture was dialyzed against MilliQ water for 7 days and lyophilized to afford CNC (+) (400 mg) as a fluffy white solid.

Elemental Analysis: C 40.15%, H 5.91%, N 0.53%, S 0.00%. ζ-potential: (48.91 ± 3.81) mV.

### 4.4. Preparation of VP-CNC Nanoformulations

VP-CNC(−) and VP-CNC(+) 10:1 (100 mg: 10 mg) were used. In a 5 mL stainless steel jar, sulfated CNC (100 mg) or CNC (+) (100 mg) and VP (10 mg) were added. The powders were grinded at 10 Hz for 15 min using three stainless steel balls (∅ = 0.5 cm).

VP-CNC(+) ζ-potential: (+36.03 ± 2.91) mV; VP-CNC(−) ζ-potential: (−34.69 ± 3.13) mV.

### 4.5. BV2 Cell Culture

BV2 murine immortalized microglial cells (mouse, C57BL/6, brain, microglial cells, Tema Ricerca, Genova, Italy; 16–20 passages) were thawed and kept in culture in a 75 cm^2^ flask in a medium containing RPMI with the addition of 10% of heat-inactivated (56 °C, 30 min) fetal bovine serum (FBS, Gibco, Milan, Italy), 1% glutamine, and a 1% penicillin–streptomycin solution (Merck, Darmstadt, Germany). Cells were cultured at 37 °C and 5% CO_2_ with daily medium change until confluence (70–80%). Trypan blue staining was used for cell counting.

### 4.6. Treatments

VP was dissolved in a vehicle composed of bidistilled water and DMSO (1:1) to obtain a homogeneous 1 mg/mL dispersion. The dispersion was then diluted with RPMI to obtain final concentrations of 0.1, 1, 10, and 100 mg/mL. VP-CNC(−) and VP-CNC(+) were dissolved in bidistilled water to obtain a 1 mg/mL solution, corresponding to 100 µg/mL of VP. The VP-CNC solutions were then diluted with RPMI to obtain concentrations of 0.1, 1, 10, and 100 µg/mL, corresponding to 0.01, 0.1, 1, and 10 µg/mL of VP, respectively. Cells were treated with vehicle or VP, VP-CNC(−), and VP-CNC(+) for 4 h, and then, to induce neuroinflammation, BV2 cells were stimulated for 24 h with a bacterial lipopolysaccharide from Gram- (LPS, 250 ng/mL Merck, Darmstadt, Germany).

### 4.7. Sulforhodamine B (SRB) Assay

Cell viability was assessed by the sulforhodamine B (SRB) assay [56]. Cells (2 × 10^4^ cells in 200 mL) were seeded in 96-well plates incubated with vehicle, VP, VP-CNC(−), and VP-CNC(+) (0.1–1–10–100 μg/mL) in the presence or absence of LPS stimulation. Cells were fixed in 50% trichloroacetic acid at 4 °C for 1 h, treated with a solution of SRB 4 mg/mL in 1% acetic acid and incubated for 30 min at room temperature. Wells were washed four times with 1% acetic acid, added with 200 mL of TRIS HCl solution (pH 10) and incubated for 5 min with shaking. Absorbance was determined using a microplate reader at 570 nm. All treatments were carried out in six technical replicates across three independent experiments, and cell viability was expressed relative to the mean value of the control group.

### 4.8. Cell Counting and Morphology

Cell counting and measurement of the cell diameter and soma surface area were performed by experimenters who were blind to the cell culture conditions on images taken by a Leica DM IL LED FLUO optical microscope and analyzed through the ImageJ 2 14.0 program, used for the quantification of the total cell number, soma diameter, and area. The pro-inflammatory state was assessed by evaluating the presence of elongated processes, increased soma cell size, and an overall shift from a rounded shape (resting condition) to a spindle-shaped or multipolar-shaped (proinflammatory state) phenotype. Next, the ratio of “pro-inflammatory” cells to the total number of cells was calculated for each photo. Finally, a measurement of cell length, expressed in µm and calculated using ImageJ 2.14 software, was conducted, which allowed for better discrimination of the inflammatory phenotype (non-pro-inflammatory, approximately 20 µm; pro-inflammatory > 40 μm). The cells were counted per mm^2^ microscopic area in at least ten randomly selected fields. For each treatment group, three independent experiments were performed [57].

### 4.9. Western Blot Analysis

BV2 cells were lysed using a lysis buffer. The insoluble pellet was separated by centrifugation (12,000× *g* for 30 min, 4 °C), and the total protein concentration in the supernatant was measured using the Bradford colorimetric method (Merck, Milan, Italy) [56]. Protein samples (20 μg) were separated on 10% SDS-PAGE [58] and then blotted onto Midi Nitrocellulose membranes using a Trans-Blot Turbo Transfer Starter System (Bio-Rad Laboratories, Milan, Italy). Blots were incubated overnight at 4 °C with primary antibodies against pERK1/2 (1:1000, Cell Signaling Technology, Danvers, MA, USA), SIRT1 (1:1000, Santa Cruz Biotechnology, Dallas, TX, USA), NFκB p65 (1:1000, Santa Cruz Biotechnology), and p-NFκB p65 (1:500, Santa Cruz Biotechnology). After being washed with PBS containing 0.1% Tween, the nitrocellulose membranes were incubated with goat anti-rabbit or anti-mouse horseradish peroxidase-conjugated secondary antibodies (1:3000, Jackson ImmunoResearch Labs, West Grove, PA, USA) for 2 h at room temperature (RT; 20–22 °C). After washing, blots were developed using an enhanced chemiluminescence detection system (ChemiDoc Imaging Systems, Bio-Rad, Milan, Italy), and signal intensity (pixels/mm^2^) was quantified using ImageJ 2.14 (NIH, Bethesda, MD, USA). Exposure and development times were standardized for all blots. For each sample, signal intensity was normalized to GAPDH (1:1000, Santa Cruz Biotechnology), and the acquired images were quantified using ImageJ 2.14 software.

### 4.10. Statistical Analysis

The results are expressed as mean ± SEM. A one-way analysis of variance (ANOVA) followed by the Tukey post hoc test was used for statistical analysis. Student’s *t* test was used when necessary. Values of *p* < 0.05 were considered significant. Outliers were identified and excluded from each experimental set using the ROUT method [59]. The software GraphPad Prism version 10.6.0 (GraphPad Software, San Diego, CA, USA) was used in all statistical analyses.

## 5. Conclusions

This study aimed to investigate the anti-neuroinflammatory properties of *Vitis vinifera* L., the common grapevine, a valuable source of antioxidant bioactive molecules such as stilbenes (e.g., resveratrol and viniferin) and flavonoids. However, the substantial variability of conventional *V. vinifera* extracts limits the production of standardized derivatives with consistent metabolite profiles. To obtain standardized products, technologies capable of ensuring reproducible metabolite production, eliminating seasonal and geographical variability, and improving environmental sustainability while reducing contamination risks are required. For this purpose, we employed in vitro plant cell culture technology to generate uniform, contaminant-free plant material. Additionally, to address the common issue of poor bioavailability of natural products, we developed a water-soluble CNC-based formulation of the phytocomplex.

We found that both the characterized plant cell culture-derived *V. vinifera* phytocomplex and its CNC formulation attenuated microglia-mediated neuroinflammation by reducing the proinflammatory phenotype, preserving cell viability, suppressing NF-κB activation and ERK1/2 phosphorylation, and enhancing SIRT1 expression.

These findings confirm the therapeutic potential of *V. vinifera* L. as an anti-neuroinflammatory intervention, underscore the value of plant cell culture technology for producing standardized and reproducible phytocomplexes, and highlight CNC as a safe and innovative delivery system. Overall, our results support biotechnology-driven strategies to improve the consistency and efficacy of natural products for potential clinical applications in neuroinflammatory and neurodegenerative conditions.

## Data Availability

The data presented in this study are available upon request from the corresponding author.

## Supplementary Materials

The following supporting information can be downloaded at: https://www.mdpi.com/article/10.3390/molecules31010196/s1, Table S1: Main compounds tentatively identified in *Vitis vinifera* V1 by LC–MS^n^ (negative ion mode).

## Abbreviations

The following abbreviations are used in this manuscript:

AKT	activation of protein kinase B
CNC	cellulose nanocrystal
CNS	central nervous system
CSF1R	colony-stimulating factor 1 receptor
ERK	extracellular signal-regulated kinases
LPS	bacterial lipopolysaccharide from Gram-
MAPK	mitogen activated protein kinase
NF-κB	nuclear factor κB
RNS	reactive nitrogen species
ROS	reactive oxygen species
SIRT1	silent information regulator sirtuin 1
TLR4	toll-like receptor 4
VP	*Vitis vinifera* L. phytocomplex
