# Supplementary material for: Viniferin-Rich Phytocomplex from Vitis vinifera L. Plant Cell Culture Mitigates Neuroinflammation in BV2 Microglia Cells
Source: Molecules. 2026 Jan 5;31(1):196. doi: 10.3390/molecules31010196 (PMC12787426; doi:10.3390/molecules31010196)
Supplement: Supplementary file 1 [file molecules-31-00196-s001.zip › molecules-4045258-supplementary.pdf]

Viniferin-Rich Phytocomplex from Vitis vinifera L. Plant Cell Culture Mitigates Neuroinflammation in BV2 Microglia Cells

Giacomina Videtta, Chiara Sasia, Sofia Quadrino, Oriana Bertaiola, Chiara Guarnerio, Elisa Bianchi, Giacomo Biagiotti, Barbara Richichi, Stefano Cicchi, Giovanna Pressi and Nicoletta Galeotti

Supplementary Material

Table S1. Main compounds tentatively identified in *Vitis vinifera* V1 by LC–MS^n (negative ion mode)

n.	RT (min)	Ion	m/z (measured)	Main MS^n fragments (m/z)	Tentative identification
1	5.8	$[M–H]^-$	551	389, 227	Resveratrol dihexoside
2	7.85	$[M–H]^-$	939	777, 615, 453	Viniferin trihexoside
3	8.50	$[M–H]^-$	939	777, 615, 453	Viniferin trihexoside (isomer)
4	8.62	$[M–H]^-$	939	777, 615, 453	Viniferin trihexoside (isomer)
5	9.8	$[M–H]^-$	389	227	Resveratrol hexoside
6	10.2	$[M–H]^-$	939	777, 615, 453	Viniferin trihexoside (isomer)
7	11.6	$[M–H]^-$	777	615, 453	Viniferin dihexoside
8	11.6	$[M–H]^-$	777	615, 453	Viniferin dihexoside (isomer)
9	11.9	$[M–H]^-$	777	615, 453	Viniferin dihexoside (isomer)
10	12.9	$[M–H]^-$	809	647, 485	Unknown stilbenoid derivative
11	12.9	$[M–H]^-$	839	676, 513	Unknown stilbenoid derivative
12	13.0	$[M–H]^-$	777	615, 453	Viniferin dihexoside
13	15.0	$[M–H]^-$	615	453	Viniferin hexoside
14	15.0	$[M–H]^-$	676	515	Unknown
15	15.6	$[M–H]^-$	645	485	Unknown
16	17.0	$[M–H]^-$	515	353, 333, 274, 215	Unknown
17	17.6	$[M–H]^-$	453	436, 411, 369, 333	Viniferin
